# Naringin Ameliorates Monocrotaline-Induced Pulmonary Arterial Hypertension Through Endothelial-To-Mesenchymal Transition Inhibition

**DOI:** 10.3389/fphar.2021.696135

**Published:** 2021-07-15

**Authors:** Yonghui Wu, Changhong Cai, Yijia Xiang, Huan Zhao, Lingchun Lv, Chunlai Zeng

**Affiliations:** Department of Cardiology, Lishui Hospital of Zhejiang University, The Fifth Affiliated Hospital of Wenzhou Medical University, Lishui Municipal Central Hospital, Lishui, China

**Keywords:** naringin, pulmonary arterial hypertension, endothelial-to-mesenchymal transition, monocrotaline, endothelial cell, rat

## Abstract

Pulmonary arterial hypertension (PAH) caused by enhanced arterial pressure increases vessel resistance in the lung. Endothelial-to-mesenchymal transition (EndMT) plays key roles in the vascular remodeling in PAH. Naringin, a protective gaseous mediator is commonly extracted from tomatoes and citrus fruits (such as grapefruits), and demonstrates anti-inflammation, anti-oxidant, anti-proliferation, and anti-tumor effects. Meanwhile, the association of Naringin and the process of EndMT is still unclear. In this study, monocrotaline (MCT) administration (60 mg/kg) was delivered for the induction of PAH in rats. Following this, Naringin (concentrations: 25, 50, and 100 mg/kg/day) was used for treatments. Human Umbilical Vein Endothelial Cells (HUVECs) were stimulated with Naringin and transforming growth factor β1 (TGFβ1, 10 ng/ml). As the result, Naringin was demonstrated to inhibit EndMT and alleviate PAH progression. In particular, in HUVECs, Naringin significantly suppressed the mesenchymal marker expression induced by TGFβ1 treatment, enhanced the endothelial marker expression, and inhibited the activation of ERK and NF-κB signaling pathways. To conclude, this study provided novel evidence suggesting the beneficial effects of Naringin in PAH through the inhibition of the ERK and NF-κB signaling pathways and the EndMT progression in pulmonary arteries.

## Introduction

Pulmonary arterial hypertension (PAH) is caused by continued enhanced pulmonary arterial pressure (PAP) and blockages formation in small and medium-sized pulmonary arterial (PAs), ultimately leading to right ventricular hypertrophy (RVH), functional failure, and untimely death (De Jesus Perez, 2016). A large number of studies have focused on the important role endothelial cell (EC) dysfunction played in the pathogenic mechanisms of PAH, meanwhile the pathogenesis of PAH is multi-factorial (Budhiraja et al., 2004). Injury of endothelial cells potentially lead to the loss of endothelial vasoactive mediator balancing, vasoconstriction, disorders in EC proliferations and small PAs loss (Sakao et al., 2009). Abnormal EC proliferations causes the plexiform lesion formation, which is the characteristic of PAH (Derrett-Smith et al., 2013). For this reason, endothelial function improvement is potentially applicable for effective treatments of PAH.

Endothelial dysfunction causes changes in endothelial vasoactive mediators, vasoconstriction injury, endothelial cell proliferation/apoptosis imbalance and endothelium-mesenchymal transformation (EndMT), which contribute to the progression of PAH (Ranchoux et al., 2015). EndMT is featured by the loss of unique phenotype in endothelial cells, and subsequent gaining of the mesenchymal phenotypes, which are marked by the loss of connection and polarity between cells and the gaining of cellular motility and invasion (Good et al., 2015). This phenomenon is featured by the observation when ECs lost specific endothelial markers, including CD31, vascular endothelial cadherin (VE-cadherin), and Von Willebrand Factor (vWF), while progressively expressing mesenchymal markers, including fibronectin (FN), vimentin, and *α*-smooth muscle actin (*α*-SMA) (Frid et al., 2002). EndMT is reported to majorly contribute to the processes of both embryonic development and fibrotic lung disease pathogenesis (Leopold and Maron, 2016; Willis and Borok, 2007). EndMT has also been reported to play critical roles in pulmonary vascular remodeling in both patients and animals of PAH. For this reason, new therapeutic strategies targeting EndMT inhibition and endothelial function improvement are potentially applicable for the treatments of PAH.

Naringin, a flavanone -7- O–glycoside, naturally occurs in citrus fruits such as grapefruits (Burke et al., 2019). Previous studies suggest the beneficial effects of naringin supplements in preventing obesity, diabetes, and other metabolic syndromes (Alam et al., 2014). Naringin also has a variety of pharmacological properties, such as anti-oxidation, anti-inflammation, anti-mutagenesis, anticancer, antibacterial, and the lowering of cholesterol level (Wang et al., 2013). Recently, several reports have shown that naringin could inhibit inflammation in acute lung injury and carcinogenesis in mice (Kim et al., 2018; Zhang et al., 2018). Given these potentials of naringin, in this study, we mainly focused on the investigations of the effects of naringin in MCT-induced rat PAH *via* inhibitions of EndoMT and improvements of endothelial function.

## Materials and Methods

### Animals

All Sprague-Dawley rats (230–250 g, 7 weeks-old, male) were commercially purchased from the experimental animal center of Zhejiang Province. All rats were caged under a standard environment (20–26°C, 45–55% humidity, 12 h light/dark cycle, standard diet). All procedures for animal study were approved by the ethics review of animal use application of the Fifth affiliated Hospital of Wenzhou Medical University.

### Animal Model and Experimental Design

Rats were divided into five experimental groups: 1) control (*n* = 6), 2) MCT group (*n* = 10), 3) low- Naringin group (*n* = 10; 25 mg/kg/d), 4) medium- Naringin group (*n* = 10; 50 mg/kg/d) and 5) high- Naringin group (*n* = 10; 100 mg/kg/d). The PAH rat model was set up by a single dose of subcutaneous MCT injection (60 mg/kg, Sigma-Aldrich, MO, United States). Aliquots of saline were injected in the control group. According to a previous study, naringin (25, 50, and 100 mg/kg/d; MedChem Express, NJ, United States) was intragastrically administered for 14 consecutive days from day 15–28 after MCT injection in the treatment groups (Cai et al., 2019). Each Rat was weighed every week for the adjustment of administered doses.

### Hemodynamic Measurement

Right ventricle systolic pressure (RVSP) measurement was conducted as in a previous study (Wu et al., 2020). Briefly, pentobarbital sodium (60 mg/kg, Sigma-Aldrich, MO, United States) through intra-peritoneal (i.p.) injection was performed for anesthetization of the rats. A venous catheter (BioPac Systems, Inc.) connected to a pressure transducer through a tube was placed in the right ventricle (RV) through the right external jugular vein.

### Right Heart Hypertrophy Assessment

After RVSP measurement, the body weight (BW) of each rat was measured. For the measurement of RVH, pentobarbital sodium (150 mg/kg) injection was performed for the scarification of rats. Weights of both RV and left ventricle (LV) plus septum (S) were recorded. RVH were assessed using both RV/(LV + S) ratio and RV/BW ratio as gravimetric indexes.

### Morphological Analysis

Lung tissues were harvested, embedded into paraffin, and cut into slices (4 μm). Hematoxylin and eosin (H&E) staining was used for the evaluation of pulmonary arteries morphology. Five slices of pulmonary arteries with diameter ranged from 50 to 150 µm were randomly picked and assessed under microscope (Nikon, Japan; magnification, ×400). Pulmonary artery wall thickness was assessed as follows: vascular wall thickness percentage (wt%) = wall thickness/outer diameter × 100%; the percentage of vascular wall area (WA%) = wall transection area/cross-sectional area × 100%.

### Cell Culture and Treatment

HUVECs were commercially purchased and cultivated in endothelial cell medium (ScienCell) with 10% fetal bovine serum (Gibco, Carlsbad, CA) and 1% penicillin/streptomycin solution, and incubated at 5% CO2 under 37°C. Cells with passage time between 3 and 8 were subjected to the following study.

### Endothelial-to-Mesenchymal Transition of Human Umbilical Vein Endothelial Cells *in vitro*

Cells cultured with an approximate 80% confluency were ready for experiments. In order to investigate the potential effect of naringin to EndMT, HUVECs was pretreated with serum starvation overnight, followed by the treatment of TGFβ1 (10 ng/ml, PeproTech, NJ, United States) and with or without naringin at different concentrations (10, 50, and 100 μM, MedChem Express, NJ, United States) treatment for different treatment times (0, 6, 12, 24, and 48 h). EndMT was revealed by the decrease in endothelial markers (CD31 and vWF) and increase in mesenchymal markers (*α*-SMA and fibronectin) using both immunoblot analysis and Immunohistochemistry.

### Cell Proliferation Assay

The cell counting Kit-8 (CCK-8, Beyotime, Jiangsu, China) was used for the assay of cell proliferations. Cells were plated into 96-well plates (1 × 104 cells/well) and cultivated for 24 h. Cells were treated with TGFβ1 and with or without different concentrations of naringin for different treatment times. Aliquots of 10 μL CCK-8 solutions were used to suspend (2 h, 37°C) samples. Cell absorbencies were measured at 450 nm by spectrophotometer.

### Cell Scratch Test

Cells were plated into 6-well plates and cultivated for 24 h. A single scratch in each well was drawn using a 200 μL pipette tip. Cell culture was washed with PBS and then incubated in culture medium with or without TGFβ1 and naringin. Images of scratches were recorded and assessed at different time points (0, 24, and 48 h) using Olympus inverted microscope.

### Histological Analysis of Endothelial-to Mesenchymal Transition

*In vivo*, lung tissue samples were used for immunohistochemical and immunofluorescence staining. Lung slices were treated by dewaxing and rehydrating, blocked in 5% BSA, and incubated with *α*-SMA (A2547, 1:400 dilution; Sigma-Aldrich, MO, United States), vWF (ab6994, 1:200 dilution; Abcam, Cambridge, United Kingdom), and CD31 (ab24590, 1:500 dilution; Abcam, Cambridge, United Kingdom) primary antibodies (4°C, overnight) and secondary antibodies at room temperature (RT) for 30 min.

*In vitro*, HUVECs were plated on glass slides, washed with PBS, and fixed by 4% paraformaldehyde (30 min). The cells were treated with PBS containing 0.5%TritonX-100 for 20 min for permeabilizing. Primary antibodies *α*-SMA (A2547, 1:400 dilution) and CD31 (ab24590, 1:500 dilution) antibody was incubated with the glass slides (4°C, overnight). Glass slides were washed with PBS (three times) and subjected to secondary antibody incubation (1:50, Beyotime, China) for 30 min at RT. Images were captured under a microscope. Images of 3–5 visual fields were randomly selected and analyzed by ImageJ software.

### Western Blot Analysis

Lung tissues and huvec cells were lyzed using radio-immunoprecipitation assay buffer (Beyotime, Shanghai, China). Samples were centrifuged at 13,000 rpm for 10 min (4°C). Supernatants were collected. Protein concentrations were evaluated by bicinchoninic acid protein assay (Beyotime). Samples were fractionated by sodium dodecyl sulfate-polyacrylamide gel electrophoresis (SDS-PAGE) and transferred to polyvinylidene fluoride (PVDF) membranes. The membranes were then blocked in 5% BSA and incubated at 4°C overnight with primary antibodies: TGFβ1 (sc146, 1:1,000 dilution, Santa cruz Biotechnology), *α*-SMA (A2547, 1:1,000 dilution, Sigma), FN (ab6328, 1:1,000 dilution, abcam), CD31 (ab24590, 1:1,000 dilution, abcam), vWF (ab6994, 1:1,000 dilution, abcam), Vimentin (ab20346, 1:1,000 dilution, abcam), VE-cadherin (ab205336, 1:1,000 dilution, abcam), Twist (ab175430, 1:1,000 dilution, abcam), Snail (ab216347, 1:1,000 dilution, abcam), *p*-ERK (9101S, 1:1,000 dilution, CST), ERK (9102S, 1:1,000 dilution, CST), p-NF-κB (ab86299, 1:1,000 dilution, abcam), NF-κB (8242S, 1:1,000 dilution, CST), GAPDH (5174S, 1:1,000 dilution, CST). Subsequently, the membranes were washed with Tris-buffered saline/Tween (TBST) three times and incubated with anti-rabbit IgG HRP-conjugated antibody (7074S, 1:1,000, 1:1,000 dilution, CST) or anti-mouse IgG HRP-conjugated antibody (7076S, 1:1,000, 1:1,000 dilution, CST) for 1 h at room temperature, and scanned using the IBright protein Western blotting imaging system (Thermo Fisher, United States).

### Statistical Analysis

All results were expressed as mean ± SEM. One-way analysis of variance and Tukey's post-hoc test was performed by GraphPad Prism 7.0. Survival analysis was used to analyze the survival rate of rats (#, *p < 0.05 and ##, **p < 0.01).

## Results

### Naringin May Improve Survival in Rats With Pulmonary Arterial Hypertension

After 28 days, the survival rate of rats in the control group was 100%, and the survival rate of rats after MCT injection was about 60%. High-dose naringin (100 mg/kg) may improve the survival rate of rats with pulmonary hypertension (80%), but there was no statistical significance between each group (*p* > 0.05; Supplemental Materials Figure 1).

**FIGURE 1:**
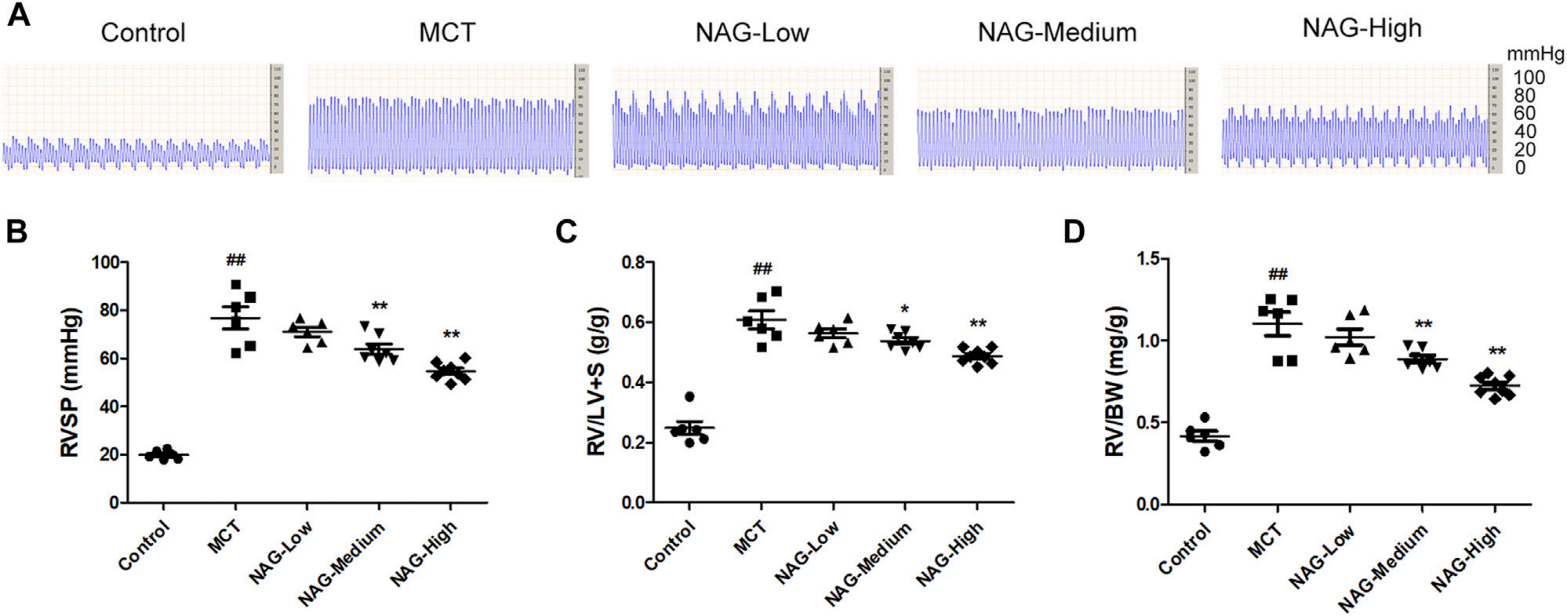
Effects of naringin on hemodynamics and RVH in the MCT-induced PAH. **(A)** Representative images of RVSP waves. **(B)** RVSP, **(C)** RV/LV + S and **(D)** RV/BW ratio. *n* = 6−8. ##*p* < 0.01 vs. control; *p < 0.05, **p < 0.01 vs. MCT; *n* = 6−8. NAG, naringin.

### Naringin Alleviated Monocrotaline-Induced Hemodynamic Changes and Right Ventricular Hypertrophy

In order to investigate the potential inhibitory effects of Naringin in the development of PAH, multiple indicators including RVSP, RV/LV + S, and RV/BW were assessed. As shown in Figures 1A,B, Administrations of MCT significantly enhanced RVSP (76.78 ± 4.20) compared to that in the control group (19.80 ± 0.64), while the increase of RVSP was markedly down-regulated by the administration of medium and high-dose of naringin (50 and 100 mg/kg) (medium: 63.90 ± 1.99; high: 54.56 ± 1.20). Meanwhile, as shown in Figures 1C,D, RV/LV + S, and RV/BW were significantly elevated after MCT administration (RV/LV + S: 0.61 ± 0.03; RV/BW: 1.10 ± 0.07), while two doses of naringin (50 and 100 mg/kg) treatments significantly recused these increases ((RV/LV + S) medium: 0.54 ± 0.01, high: 0.49 ± 0.01 (RV/BW) medium: 0.89 ± 0.02, high: 0.73 ± 0.02), thereby alleviating the right ventricular hypertrophy.

### Naringin Attenuated Monocrotaline-Induced Pulmonary Arterial Remodeling

**FIGURE 2:**
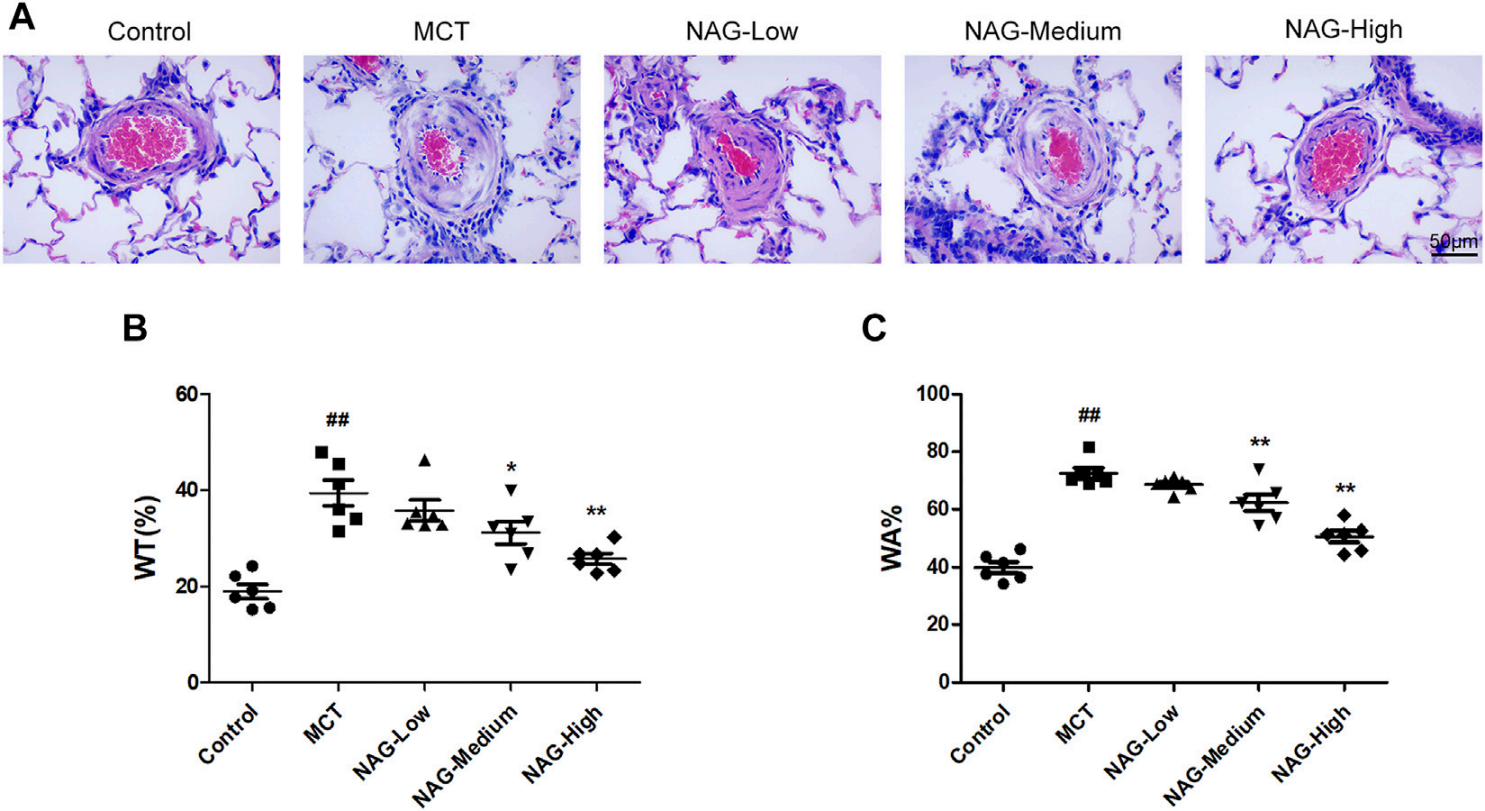
Effects of naringin on pulmonary arterial remodeling in the PAH caused by MCT administration. **(A)** Representative histopathological images of rat PAs by H&E staining. Magnification, ×400; scale bars, 50 µm. Quantifications for **(B)** wt% and **(C)** WA%. ##*p* < 0.01 vs. control; *p < 0.05, **p < 0.01 vs. MCT. *n* = 6.

For the assessments of cardiac fibrosis, histological analysis was performed based on the results of H&E staining. Subsequently, the thicknesses of pulmonary arterioles with 50–150 μm in diameter was measured. As a result, MCT administration induced significant enhancement of WT% (39.43 ± 2.44) and WA% (72.49 ± 1.76) of pulmonary arterioles. Meanwhile, both medium- (WT%: 31.16 ± 2.12; WA%: 62.35 ± 2.56) and high-dose (WT%: 25.72 ± 1.03; WA%: 50.55 ± 1.84) Naringin treatments rescued this MCT-promoted WT% and WA% increase. The results show that high-dose naringin can better improve MCT-induced PAH (Figure 2).

### Naringin Suppressed Endothelial-to-MesenchymalTransition in Monocrotaline-Induced Pulmonary Arterial Hypertension

**FIGURE 3:**
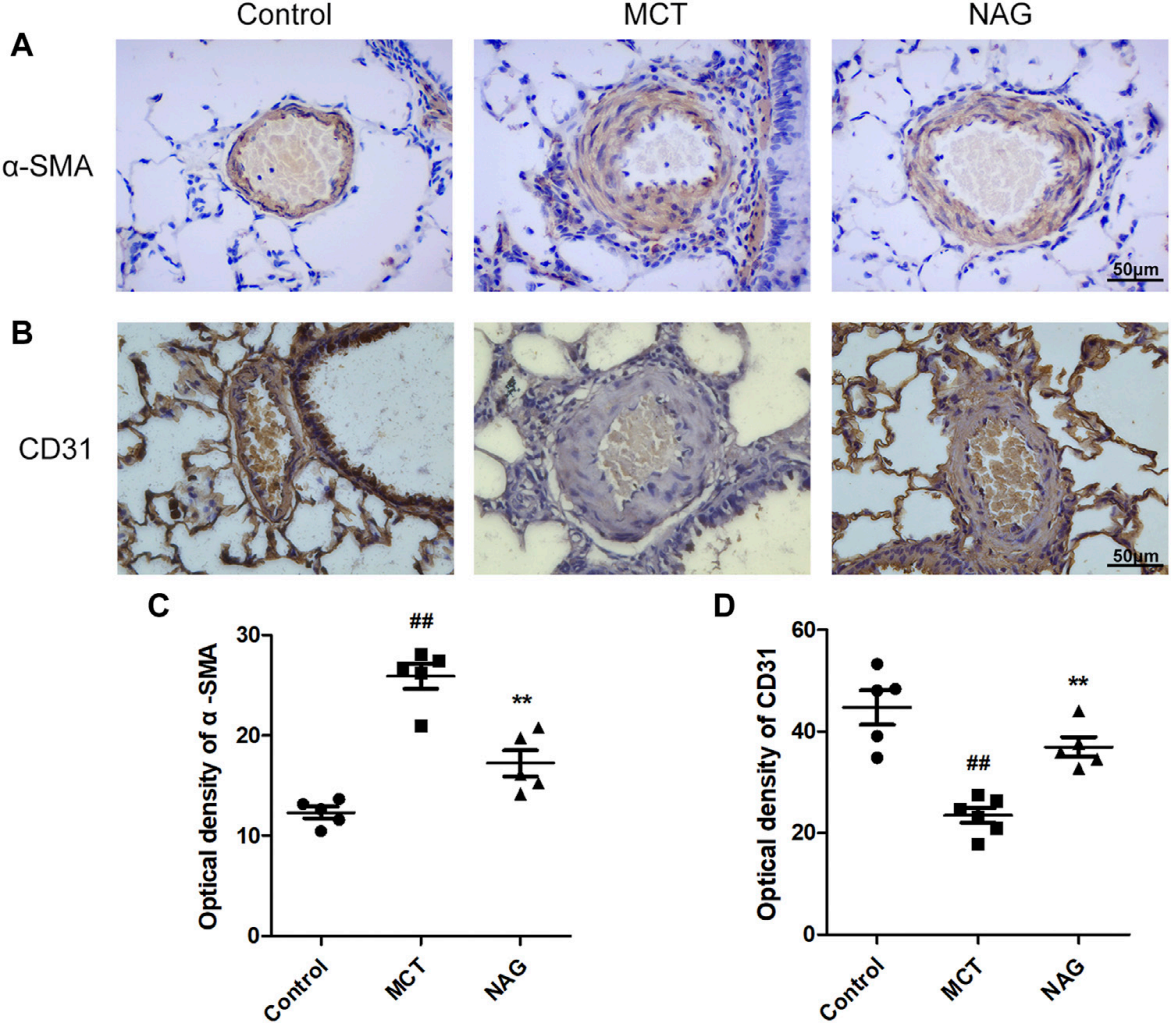
Effects of naringin on expressions of *α*-SMA and CD31 in the MCT-induced PAH. The expressions of *α*-SMA **(A)** and CD31 **(B)** in lungs in immunohistochemistry staining. Quantifications of *α*-SMA optical density **(C)** and CD31 optical density **(D)**. Magnification ×400, scale bars = 50 μm ##*p* < 0.01 vs. control; **p < 0.01 vs. MCT. *n* = 6.

**FIGURE 4:**
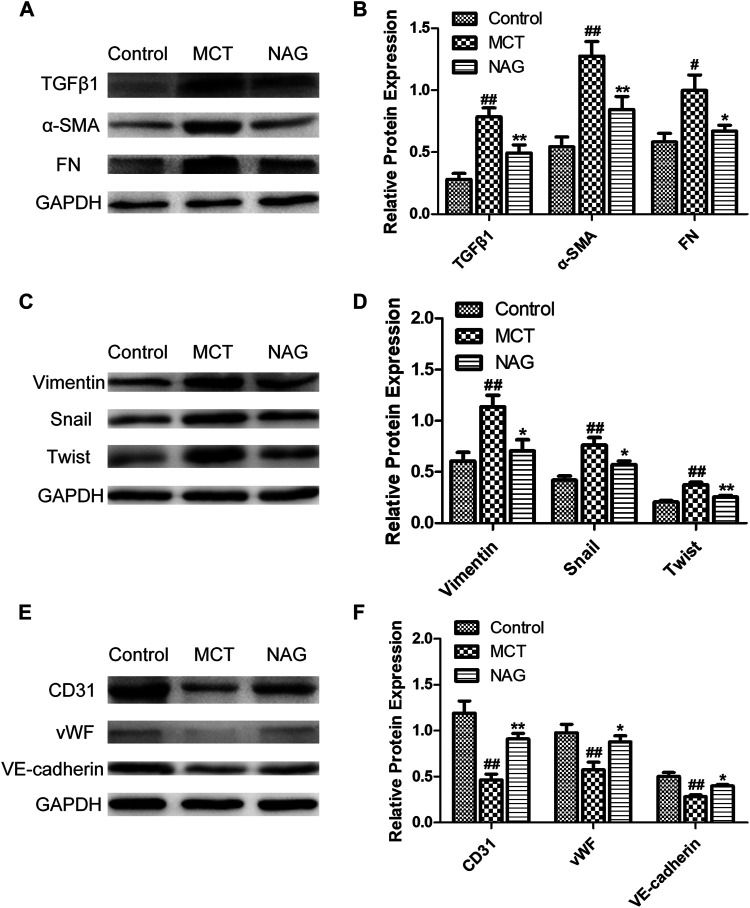
Effects of naringin on expressions of TGFβ1, *α*-SMA, FN, Vimentin, Twist, Snail, CD31, vWF, and VE-cadherin in lung tissues in rats. Expressions of TGFβ1, *α*-SMA, FN **(A,B)**, Vimentin, Twist, Snail **(C,D)** and CD31, vWF, VE-cadherin **(E,F)**. #*p* < 0.05, ##*p* < 0.01 vs. control; *p < 0.05, **p < 0.01 vs. MCT. *n* = 6.

**FIGURE 5:**
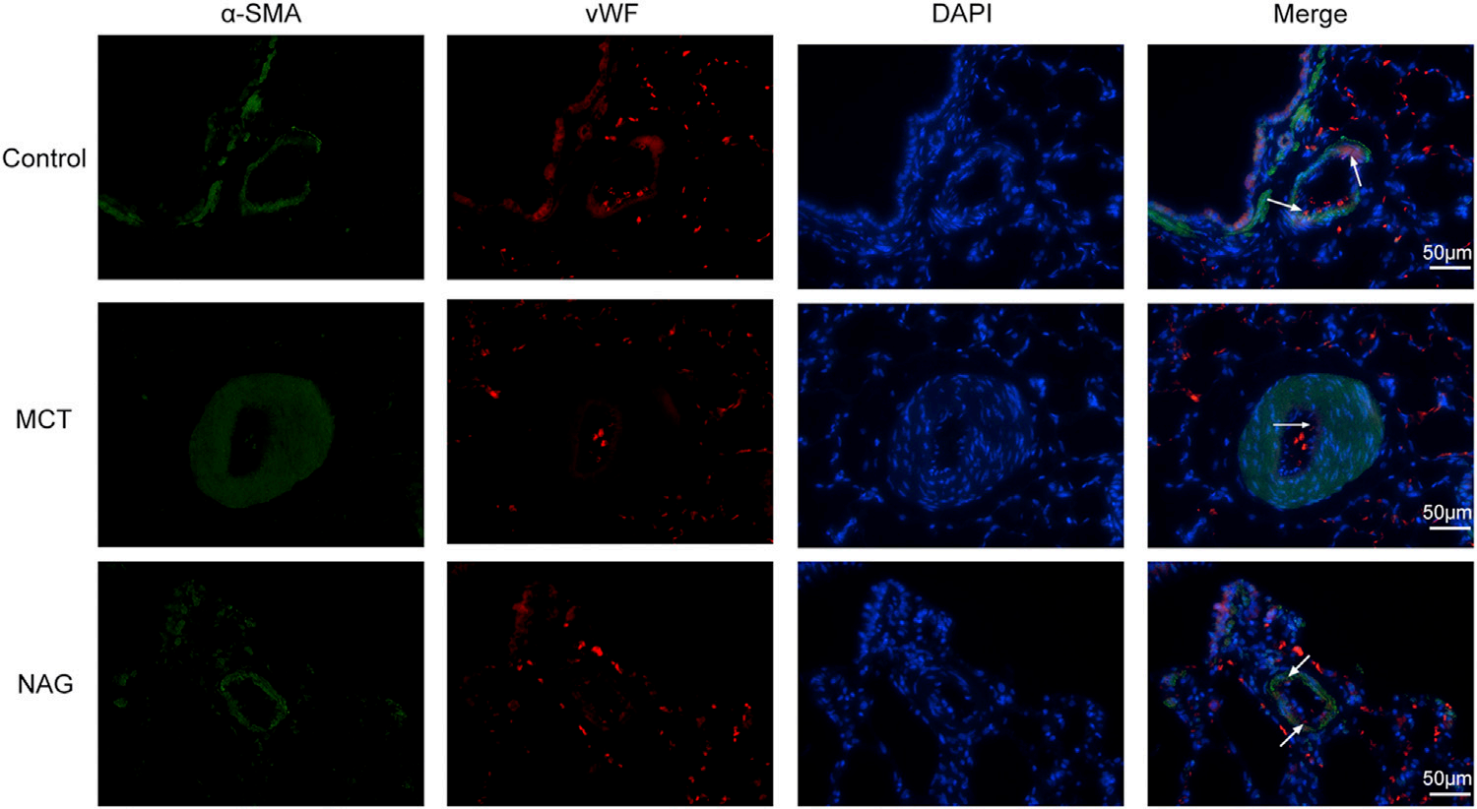
Effects of naringin on EndMT of PAH in rats was demonstrated by immunofluorescence staining. Red fluorescence represents vWF, green fluorescence represents *α*-SMA and blue fluorescence indicates DAPI nuclei staining. The white arrows in the images indicate vWF-positive cells. Magnification ×400, scale bars = 50 μm.

TGFβ1 expression changes during the development of PAH were determined by western blot. The result demonstrated that naringin rescued this up-regulation of TGFβ1 induced by MCT administration (Figures 3A,B). In previous studies, EndMT was indicated as one of the major contributors to PAH pathogenesis. As shown in Figure 3 and Figure 4, endothelial markers expressions (vWF, VE-cadherin and CD31) were downregulated, while mesenchymal markers (Vimentin, *α*-SMA and FN) and EndMT-related transcription factors (snail and twist) expressions were up-regulated in the lung samples collected from MCT-treated rats. Meanwhile, naringin treatments rescued these changes, which were further verified by western blot. The results from immunohistochemical staining were consistent with those from western blot, and we also obtained similar results by immunofluorescence staining (Figure 5), subsequently suggesting the protective effects of naringin in MCT-induced PAH through inhibiting EndMT.

### Naringin Inhibited Transforming Growth Factor β1-Induced Human Umbilical Vein Endothelial Cells Proliferation and Migration

**FIGURE 6:**
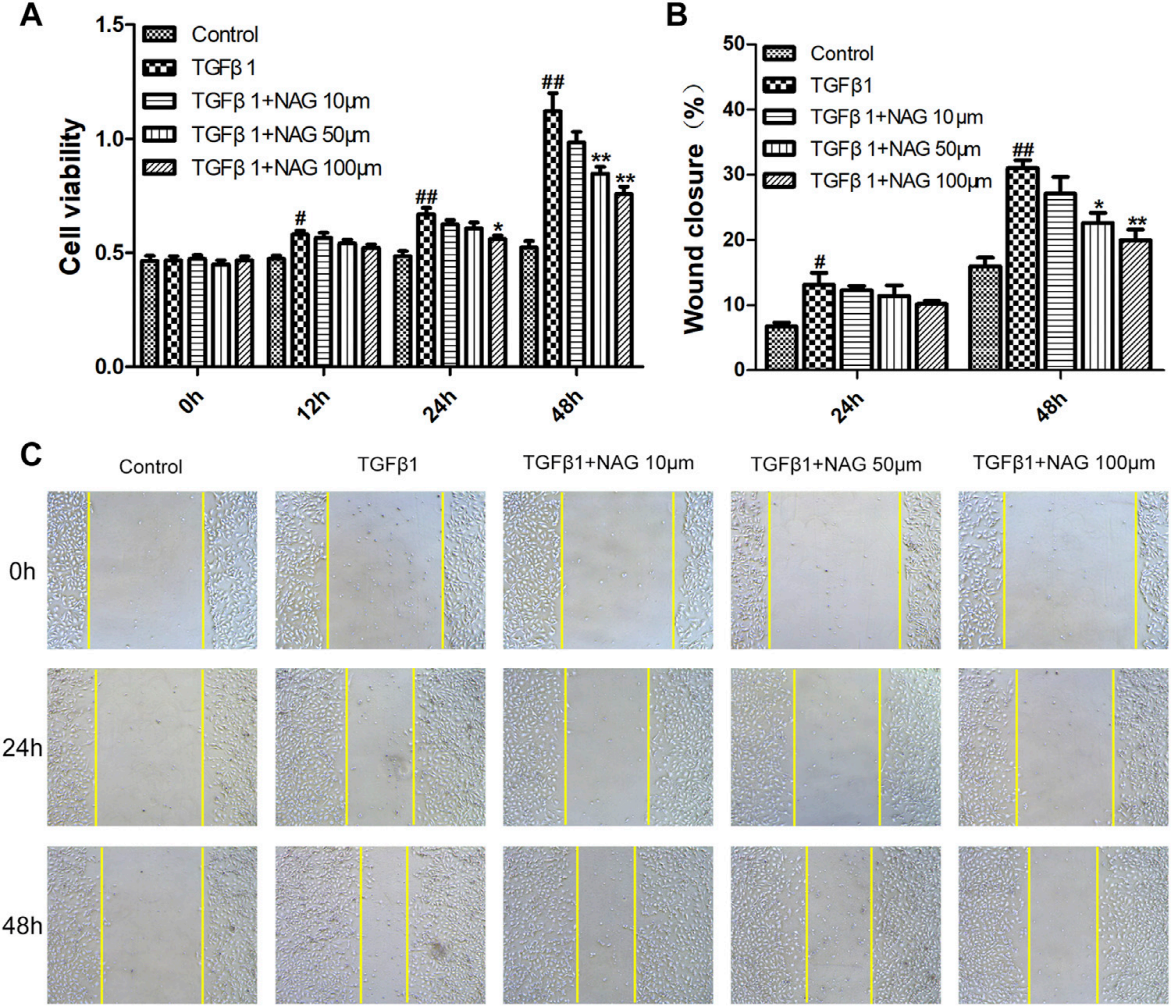
Effects of naringin on proliferation and migration of HUVECs **(A)** Serum-starved HUVECs were incubated with TGF-β1 (10 ng/ml) and the absence or presence of naringin (10, 50 and 100 μmol/L) for 0, 12, 24, and 48 h **(B,C)** Serum-starved HUVECs were stimulated with TGF-β1 (10 ng/ml) and the absence or presence of naringin (10, 50 and 100 μmol/L) for 0, 24 and 48 h #*p* < 0.05, ##*p* < 0.01 vs. control; *p < 0.05, **p < 0.01 vs. TGFβ1. *n* = 3.

To explore the effect of naringin on HUVECs proliferation, TGFβ1 and different concentrations of naringin (10, 50, 100 µM) were used to treat HUVECs for different times (0, 12, 24, and 48 h). Subsequently, the HUVECs viability were determined using CCK-8. As is shown in Figure 6A, naringin could inhibit HUVECs viability in both a dose-dependent manner and time-dependent manner.

Cell migration was analyzed by cell scratch test. Wound closure levels were increased after TGFβ1 stimulation for 24 and 48 h (Figure 6C), whereas the TGFβ1-induced migration was inhibited by naringin, suggesting that the cell proliferation and migration were significantly inhibited after 48 h of TGFβ1 stimulation. Meanwhile naringin (100 µM) significantly inhibited the cell proliferation and migration (Figure 6B). Therefore, we chose the stimulation time and concentration of TGFβ1 and naringin for the following cell experiments.

### Naringin Attenuated Transforming Growth Factor β1-Induced Endothelial-to-Mesenchymal Transition in Human Umbilical Vein Endothelial Cells

**FIGURE 7:**
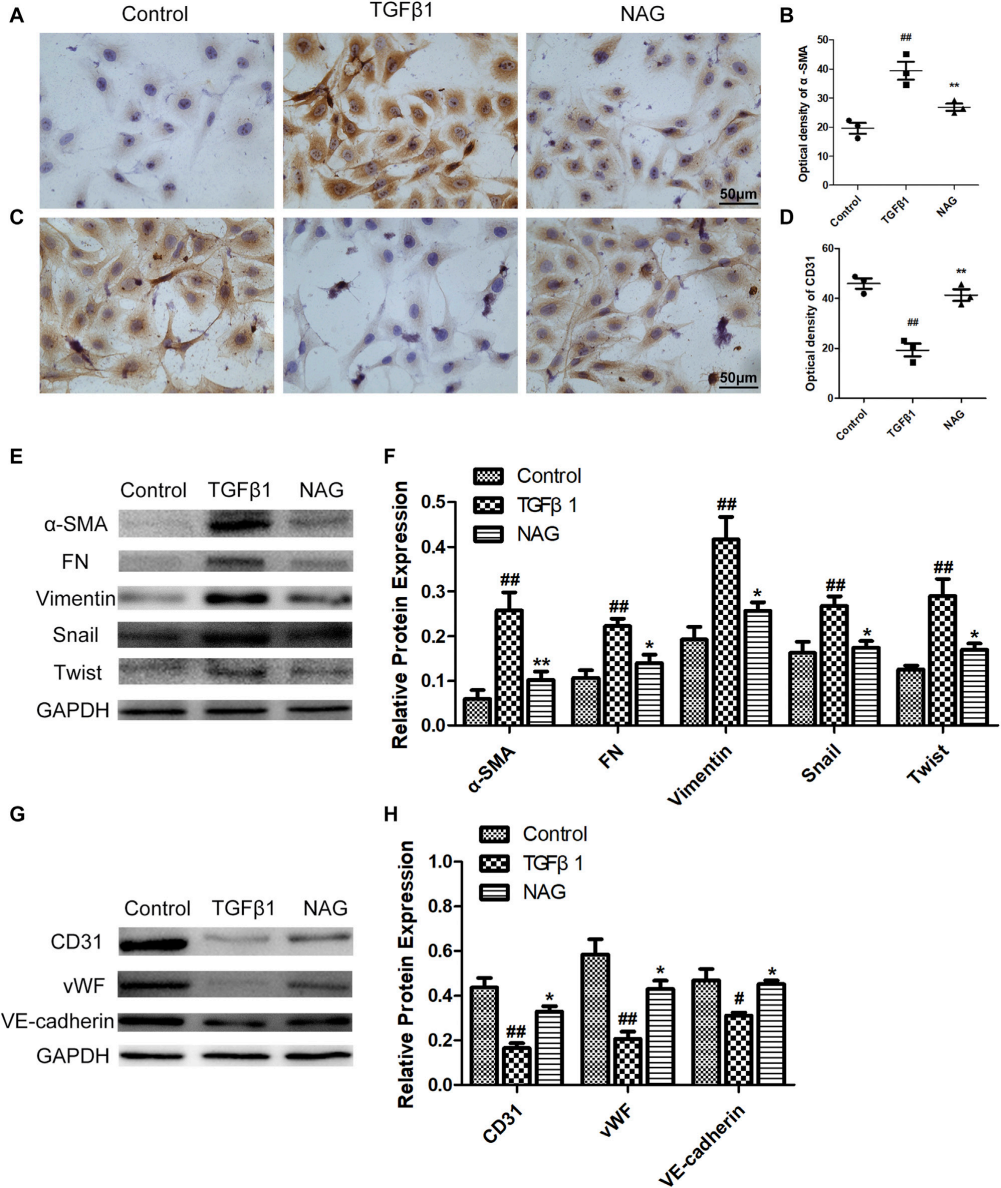
Effects of naringin on expressions of *α*-SMA, FN, Vimentin, Twist, Snail, CD31, vWF, and VE-cadherin in TGFβ1-induced HUVECs. Serum-starved HUVECs were stimulated with TGF-β1 (10 ng/ml) and with or without naringin (100 μmol/L) for 48 h. The expressions of *α*-SMA **(A)** and CD31 **(C)** in HUVECs were analyzed by immunohistochemistry staining. Quantification of *α*-SMA optical density **(B)** and CD31 optical density **(D)** were calculated. The expressions of *α*-SMA, FN, Vimentin, Twist, Snail **(E,F)** and CD31, vWF, VE-cadherin **(G,H)** were analyzed with western blot. Magnification ×400, scale bars = 50 μm ##*p* < 0.01 vs. control; **p < 0.01 vs. TGFβ1. *n* = 3.

For the investigations of the effects of naringin on EndMT, TGFβ1 was used for the induction of EndMT in HUVECs. In western blot, TGFβ1 treatments down-regulated the expressions of vWF, VE-cadherin, and CD31, while up-regulating the expressions of Vimentin, *α*-SMA, FN, snail, and twist, indicating that the process of EndMT was enhanced during this process (Figures 7E–H). These changes were validated by immunohistochemical staining, revealed by the observations that TGFβ1 significantly reduced the number of CD31 positive staining cells, while promoting the number of *α*-SMA positive cells (Figures 7A–D). However, naringin could markedly reverse TGFβ1-induced changes (Figure 7). These ffndings demonstrated that naringin attenuated TGFβ1-induced EndMT in HUVECs.

### Naringin Modulated Extracellular Regulated Kinase and Nuclear Factor-κB Signaling Pathways Activation in Transforming Growth Factor β1-Induced Human Umbilical Vein Endothelial Cells

**FIGURE 8:**
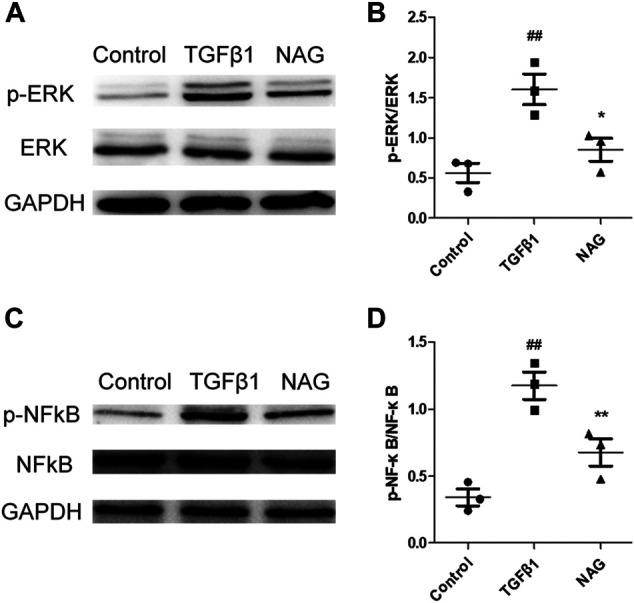
Effects of naringin on ERK and NF-κB signals. Serum-starved HUVECs were stimulated with TGF-β1 (10 ng/ml) and the absence or presence of naringin (100 μmol/L) for 48 h. Expressions of *p*-ERK, ERK **(A,B)**, *p*-NFκB, and NFκB **(C,D)**. ##*p* < 0.01 vs. control; *p < 0.05, **p < 0.01 vs. MCT. *n* = 3.

To further investigate molecular mechanisms underlying the observation that naringin recued the effects induced by TGFβ1 administration, the expressions of ERK and NF-κB signaling pathways were analyzed by western blot. Our results demonstrated that two signaling pathways were activated after TGFβ1 stimulation, while the activations were significantly blocked by naringin (Figure 8). All the clues above suggested that naringin regulated TGFβ1-induced EndMT *via* ERK and NF-κB signaling pathways.

## Discussion

In this study, we demonstrated the protective effects of naringin during the pathogenic progression of PAH in MCT-induced rat model. Naringin significantly attenuated symptoms of PAH, including pulmonary vascular remodeling and RVH. Also, our results demonstrated that naringin attenuated EndMT in PAH rat model. Furthermore, in cultured HUVECs, we also found the inhibitory role naringin played in TGFβ1-induced EndMT.

In previous studies, several animal models were widely used for novel pharmacotherapy preclinical studies of PAH. Animal models are commonly established through MCT lung injury, pulmonary hypertension caused by chronic hypoxia, and vascular endothelial growth factor receptor (VEGF-R) blockade using the tyrosine kinase inhibitor SU5416 (SU5416 plus chronic hypoxia model, SuHx model). Those different types of animal model have been intensively studied as they are able to mimic the vascular symptoms in PAH patients with severe conditions (de Raaf et al., 2014). In this study, we demonstrate decrease in survival, significant elevations in RVSP, pulmonary vascular remodeling, and RVH in PAH rat models caused by MCT administration, which are consistent with previous studies. Naringin is a flavanone glycoside synthesized from the flavanone naringenin and the disaccharide neohesperidose. Previous studies have demonstrated the potential protective effects of Naringin on diseases including osteoporosis, atherosclerosis, hypertension, and alcoholic hepatic steatosis (Zhao et al., 2020; Akintunde et al., 2020; Zhou et al., 2019). However, the efficacies of Naringin to the pathogenesis of PAH is still unknown. Here in this study, we demonstrated that naringin can improve survival and alleviate the symptoms of PAH caused by MCT administration, including diminished pulmonary vascular remodeling and RVH. Accumulating evidence has indicated that cardiac function is damaged by pulmonary interstitial fibrosis, contributing to the formation of pulmonary hypertension and causing RV failure (Andersen et al., 2019). Naringin attenuates damages in vascular endothelial caused by Oxidized low-density lipoprotein (ox-LDL) and alleviates paraquat-induced acute lung injury in mice through the suppression of collagen synthesis (Zhao et al., 2020; Chen et al., 2013). Naringenin, a conversion of naringin, can increase the protective effect of l-arginine against monocinine induced pulmonary hypertension in rats (Ahmed et al., 2014), therefore potentially participating in the protective effects of naringin on PAH. Some studies have shown that TGFβ1 can promote the proliferation and migration of endothelial cells (Wu et al., 2017; Jiang et al., 2018), while other literature has shown that TGFβ1 can reduce the proliferation and migration of endothelial cells (Baird and Durkin., 1986; Frater-Schroder et al., 1986). Thus, TGFβ1 is a special growth factor that may have two sides. Importantly, in our study, naringin is found to suppress the proliferation and migration of HUVECs induced by TGFβ1. Collectively, naringin significantly inhibited pulmonary injury and vascular endothelial injuries, therefore can be used as novel therapy for the clinical treatments of PAH.

EndMT contributes to the pathogenesis of abnormal pulmonary vascular remodeling and tissue fibrosis in PAH. As reported, some ECs are able to express both endothelial and mesenchymal phenotypes in PAH model caused by MCT administration (Good et al., 2015). EndMT is featured by the gaining of mesenchymal phenotypes and the decrease of endothelial cell markers in the endothelium (Suzuki et al., 2018). In pulmonary hypertension, EndMT causes matrix-generating fibroblasts and contributes to the progression of extracellular matrix productions and collagen depositions, and subsequently causes the pulmonary vascular remodeling (Xiong, 2015). Naringin are found to produce protective effects against End MT in atherosclerosis (Zhao et al., 2020). Here in this study, our data indicated significantly decreased endothelial markers, increased mesenchymal markers, and EndMT-related transcription factors in the lung tissue of an MCT-induced rat model of PAH. Most importantly, naringin treatments are able to rescue such alterations in PAH through the inhibition of EndMT. Our findings are further validated *in vitro* in the studies of HUVECs. Remarkably, TGFβ1-induced increase of mesenchymal markers (Vimentin, *α*-SMA and FN) and EndMT-related transcription factors (snail and twist), decrease of the endothelial markers expressions (vWF, VE-cadherin and CD31) in HUVECs are inhibited by naringin treatments. Collectively, both *in vivo* and *in vitro* studies demonstrate that naringin is able to delay the development of PAH through the inhibition of EndMT.

ERK and NF-κB signalling pathways have been reported as important factors in the maintenance of pulmonary vascular homeostasis (Cai et al., 2019; Wu et al., 2020). Increasing evidences have demonstrated the associations of ERK and NF-κB signalling pathways in the EndMT in PAH. Blockage of such pathways produces beneficial effects in PAH (Sabbineni et al., 2018; Zhang et al., 2019). In PAH patients, TGFβ1 induced EndMT vascular ECs are reported to associate with ERK and NF-κB signalling pathway activations, while activation inhibition of such signalling pathways partially recues the TGFβ1-induced EndMT in ECs (Shu et al., 2016; Zong et al., 2020). Naringin is reported to inhibit the process of osteoclastogenesis and bone resorptions through the inhibitory effects against ERK and NF-κB signalling pathway activations (Ang et al., 2011). Also, naringin is able to attenuate endothelial injuries and ox-LDL-induced EndMT (Zhao et al., 2020). In this study, consistent with previous reports, we find that TGFβ1 significantly increased the phosphorylation levels of ERK and NF-κB signalling pathway in HUVECs, which was partially reversed by naringin. Collectively, our results demonstrate that ERK and NF-κB signalling pathways are associated with the process of naringin inhibiting the TGFβ1-induced EndMT.

In conclusion, in this study, our data suggest that naringin can be used as a potential agent for the treatment of PAH, evidenced by the observation that naringin alleviates pulmonary vascular remodeling and RVH in MCT-induced PAH rats. This effect is potentially achieved through the improvement of EndMT via inhibiting ERK and NF-κB signalling pathways.

## Data Availability

The raw data supporting the conclusion of this article will be made available by the authors, without undue reservation, to any qualified researcher.
